# Successful contemporary reverse controlled antegrade and retrograde subintimal tracking without contrast medium: a case report

**DOI:** 10.1186/s13256-018-1918-2

**Published:** 2018-12-27

**Authors:** Satoshi Higuchi, Yusuke Miura, Yoshio Nishina, Kohei Koyama, Ken Kongoji, Kenichi Matsushita, Kyoko Soejima

**Affiliations:** Division of Cardiology, Department of Internal Medicine II, Kyorin University School of Medicine, 6-20-2 Shinkawa, Mitaka City, Tokyo 181-0004 Japan

**Keywords:** Reverse CART, Percutaneous coronary intervention, Chronic total occlusion, Contrast medium, Chronic kidney disease, Contrast-induced acute kidney injury, Retrograde approach

## Abstract

**Background:**

Contrast-induced acute kidney injury is one of the common adverse events related to percutaneous coronary intervention and a predictor for worse outcome. In the setting of percutaneous coronary intervention for chronic total occlusion, large amounts of contrast medium, more than 200–400 mL, are generally injected. A higher dose of contrast medium causes contrast-induced acute kidney injury more frequently. Therefore, patients who undergo chronic total occlusion-percutaneous coronary intervention are at risk for contrast-induced acute kidney injury.

**Case presentation:**

We present the case of a 77-year-old Japanese man with post-acute myocardial infarction angina pectoris, heart failure, and chronic kidney disease who underwent percutaneous coronary intervention for chronic total occlusion in his right coronary artery. In the procedure, the retrograde wire was a visible penetration mark that made contrast medium unnecessary. Contemporary reverse controlled antegrade and retrograde subintimal tracking was successfully achieved and stents were implanted without contrast medium. Contrast medium was injected two times after stent implantation to confirm coronary flow and no perforation. The total amount of contrast medium was only 8 mL for chronic total occlusion-percutaneous coronary intervention.

**Conclusion:**

Chronic total occlusion-percutaneous coronary intervention with contemporary reverse controlled antegrade and retrograde subintimal tracking without contrast medium may be safe and feasible in selected patients.

**Electronic supplementary material:**

The online version of this article (https://doi.org/10.1186/s13256-018-1918-2) contains supplementary material, which is available to authorized users.

## Introduction

Contrast-induced acute kidney injury (CI-AKI) is one of the common adverse events related to percutaneous coronary intervention (PCI) and a predictor for worse outcome. CI-AKI can develop in patients with mildly impaired kidney function as well as those with severely impaired kidney function. There are a few preventive measures such as normal saline administration and reduction of dose of contrast media. However, such preventive measures have some limitations. Normal saline poses a potential risk for worsening congestion in patients with heart failure. The feasibility of contrast medium reduction depends on lesion complexity. Ali *et al.* reported on imaging-guided and physiology-guided PCI without contrast medium [1]. Notably, this study excluded patients with chronic total occlusion (CTO). In the setting of CTO-PCI, large amounts of contrast medium, generally more than 200–400 mL, are injected [2, 3]. Therefore, patients who undergo CTO-PCI are at risk of CI-AKI. Imaging-guided and physiology-guided PCI alone would not achieve a non-use contrast medium strategy because the main problem of CTO-PCI is wire-crossing in a CTO lesion. Most of the contrast medium is injected to confirm a positional relation between the guidewire and far end of the CTO lesion before guidewire passage. Since controlled antegrade and retrograde subintimal tracking (CART) was proposed in 2006 [4], the success rate of CTO-PCI has increased. The technique can contribute to reduction/omission of contrast medium because the retrograde wire is a potential landmark, toward which the antegrade wire should pass. The present case report describes contemporary reverse CART without contrast medium.

## Case presentation

**Fig. 1:**
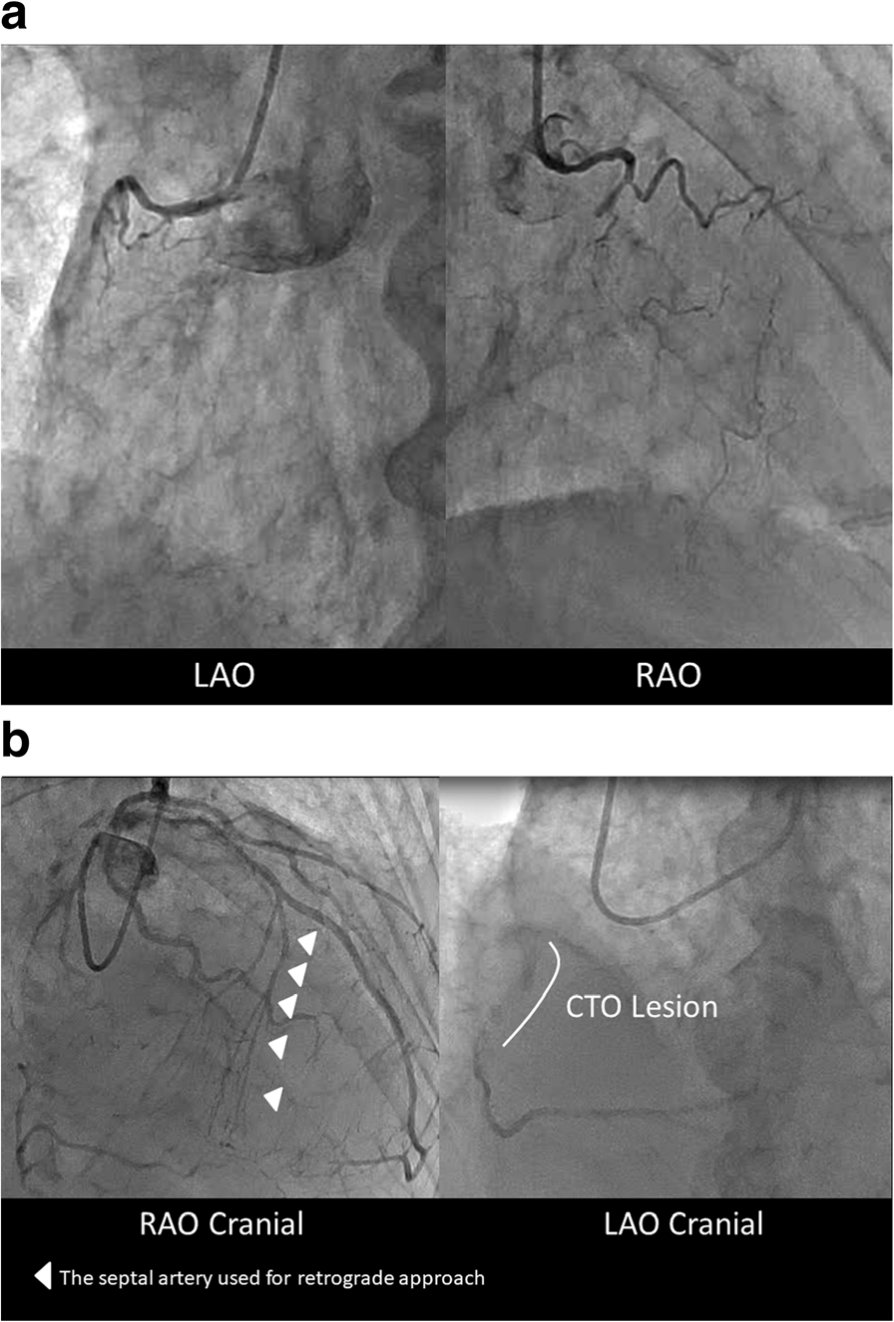
**a** Chronic total occlusion of the right coronary artery. **b** Collateral channel from the septal arteries. *CTO* chronic total occlusion, *LAO* left anterior oblique, *RAO* right anterior oblique. The arrowheads indicate the septal artery used for retrograde approach

A 77-year-old Japanese man with a history of hypertension and diabetes mellitus developed chest compression at rest and was referred to our hospital. On initial examination, we observed higher blood pressure, normal heart rate, tachypnea, and normal body temperature (154/94 mmHg, 80 beats per minute, 22 breaths per minute, and 36.6 °C, respectively). His first and second heart sounds had normal loudness, and a fourth heart sound was heard. Neither the third heart sound nor murmur was heard. A coarse crackle was heard in the lower field of his right lung. Electrocardiography (ECG) showed regular sinus rhythm and ST elevation in precordial leads. His value of troponin-I was 1682 pg/ml. The findings of the physical examination, ECG, and a high value of troponin-I supported the diagnosis of ST segment elevation myocardial infarction, classified into Killip classification **II**. Coronary angiography showed 90% stenosis of his proximal left anterior descending artery (LAD), 75% diffuse stenosis of his proximal right coronary artery (RCA), and total occlusion of his mid RCA with a Multicenter CTO Registry of Japan (J-CTO) score of 3, which originated from the septal branch (Fig. 1a and b). A month after PCI of his LAD, PCI of his RCA-CTO was planned because of remaining chest discomfort on effort.

**Fig. 2:**
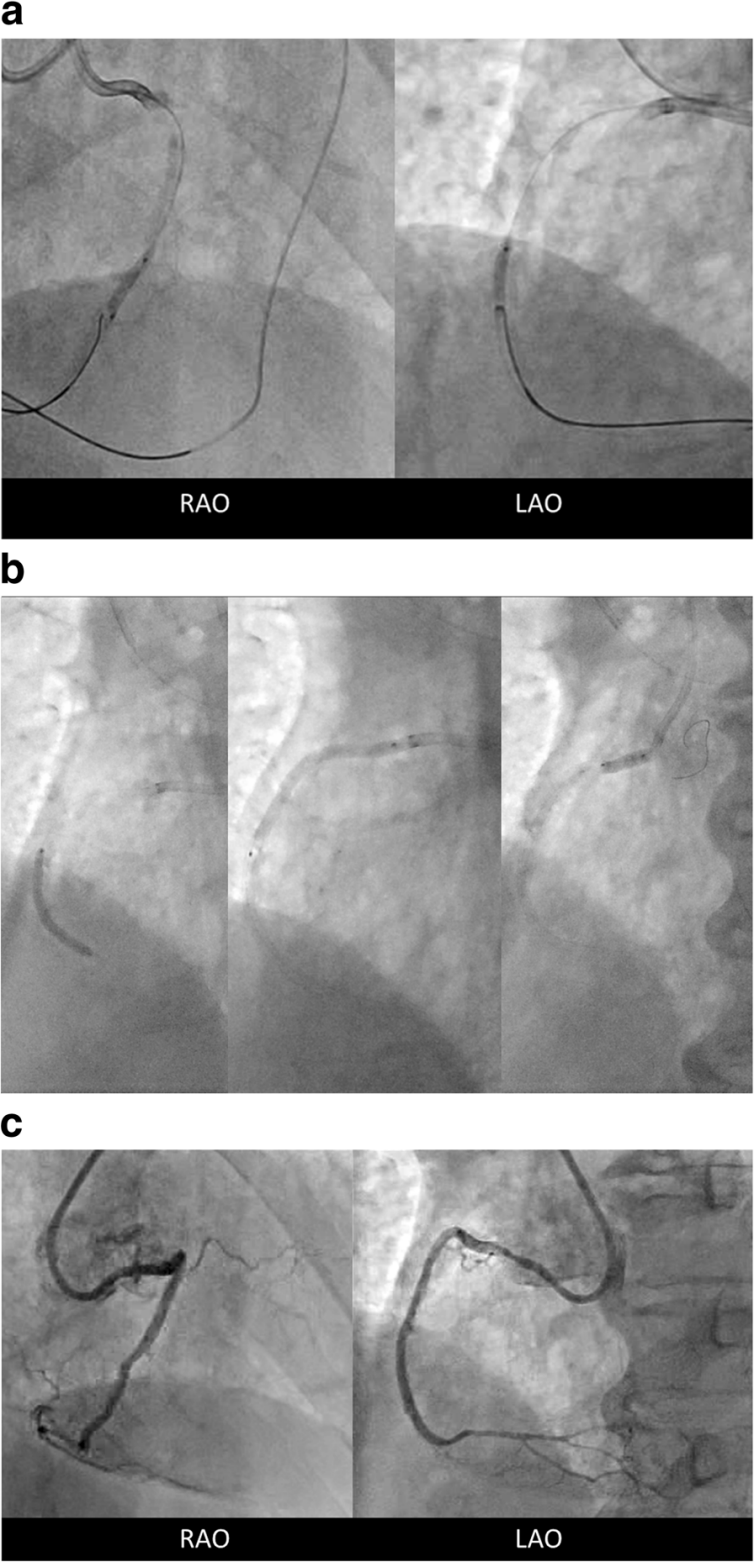
**a** Retrograde wire was to penetrate the lesion in which an antegrade balloon was dilated. **b** Stent implantation. **c** Coronary angiogram after stent implantation. *LAO* left anterior oblique, *RAO* right anterior oblique

In the second intervention, his serum creatinine level was 88 umol/L, and the stage of chronic kidney disease was 3A. His B-type natriuretic peptide level was 400 ng/L and left ventricular ejection fraction was 45%. Heart failure was compensated. Seven-French Amplatz 1.0 with side hall and 6-French Extra Backup 3.5 with side hall were engaged to his RCA and LAD, respectively. Septal channel tracking was performed with SUOH 03® supported by Caravel® in the manner of channel surfing. After the wire was advanced to the distal cap of the CTO lesion, Gaia Next 1® supported by Corsair® penetrated the lesion antegradely with the kissing wire technique; however, the antegrade wire was repelled by severe calcification and penetrated into the pseudolumen. Gaia Next 1® was changed to Grand Slam®. Then, a 2.5-mm balloon was delivered to the lesion and dilated. Gaia Second® was retrogradely advanced to the tip of the dilated balloon (contemporary reverse CART) (Fig. 2a). After intravascular ultrasound (IVUS) confirmed the retrograde wire was located in the true lumen, the wire was inserted into the antegrade guiding catheter. After externalization with RG3®, the CTO lesion was dilated with a 2.5-mm balloon. Stents were deployed from the mid to the ostium in the RCA without contrast medium, using IVUS marking and dummy wire (Fig. 2b). The ostium of the RCA was fully covered with the stent, and several struts protruded to the aorta, which was confirmed by IVUS. Contrast medium was injected two times after stent implantation to confirm coronary flow and no perforation (Fig. 2c). The total amount of contrast medium was only 8 mL for CTO-PCI. Our patient did not develop CI-AKI and his chest discomfort has disappeared (Additional file 1).

## Discussion

The present case indicated the potential feasibility of CTO-PCI without contrast medium, although we injected a small amount to confirm coronary flow and perforation after the procedure. The retrograde wiring technique confirms successful and effective CTO-PCI because stents are usually implanted in the true lumen in the far end of the CTO lesion, which is different from the antegrade approach alone. Furthermore, the retrograde wire is a visible penetration mark that makes contrast medium unnecessary. The most important tips for the present procedure are to increase success rate of retrograde wire operation and to ensure safety.

Prior to PCI, interventionists should seek promising/possible collateral channels carefully. Right anterior oblique caudal and cranial views are especially useful to find out promising/possible septal channels. Even if any visible collateral channels would be inappropriate for a retrograde approach, channel surfing could yield successful channel tracking in invisible collateral arteries at the time. Once a retrograde wire reaches a distal cap, contrast medium is unnecessary for an antegrade wire operation. In general practice, antegrade and retrograde wiring technique in a CTO lesion does not need contrast medium. IVUS-guided reverse CART contributes to procedural success and safety [5]. After externalization, IVUS can be used to detect a lesion and mark stent implantation instead of angiography. In the setting of CTO without a useful retrograde channel, ipsilateral injection may be necessary to confirm whether the wire was advanced to the true lumen and avoid wire perforation. To achieve a safe procedure, the presence of a non-epicardial channel is preferable because channel surfing in epicardial channels poses a risk for perforation and cardiac tamponade. These are the limitations regarding our strategy.

CTO-PCI was reported to be associated with severe complications such as coronary perforation, myocardial infarction, and cardiogenic shock [6]. Regarding safety, operators must be concerned about no/slow reflow, wire perforation, and cardiac tamponade. A guidewire with a soft tip seems to be appropriate to reduce a risk of wire perforation. These complications can be monitored without contrast medium. First, ST-T changes on the electrocardiogram help us determine whether coronary flow is intact. Furthermore, it would be possible to detect no/slow flow from blood speckle changes in IVUS finding. Second, wire perforation might be detected by abnormal wire motion which is not running along the coronary vessel, development of frequent premature ventricular contraction, or the touch of the operator’s hand. Finally, acute pericardial effusion can be detected by observing whether the heart shadow on the left anterior oblique view moves. The motion of the cardiac border disappears prior to development of cardiac tamponade. These identification methods are simple, useful, and reliable for a safe procedure.

## Conclusion

CTO-PCI with reverse CART without contrast medium may be safe and feasible in selected patients. This strategy can be a boon for patients with impaired kidney function and/or congestive heart failure susceptible to hydration.

## Additional file

Additional file 1: Timeline of the Clinical Course. (DOCX 15 kb)

## Abbreviations

CART: Controlled antegrade and retrograde subintimal tracking
CI-AKI: Contrast-induced acute kidney injury
CTO: Chronic total occlusion
ECG: Electrocardiography
IVUS: Intravascular ultrasound
LAD: Left anterior descending artery
PCI: Percutaneous coronary intervention
RCA: Right coronary artery
