# Supplementary material for: Successful contemporary reverse controlled antegrade and retrograde subintimal tracking without contrast medium: a case report
Source: J Med Case Rep. 2018 Dec 27;12:390. doi: 10.1186/s13256-018-1918-2 (PMC6307242; doi:10.1186/s13256-018-1918-2)
Supplement: Supplementary file 1 — Timeline of the Clinical Course. (DOCX 15 kb) [file 13256_2018_1918_MOESM1_ESM.docx]

Timeline

| Dates | Relevant Past Medical History and Interventions | | | | |  |  |  |  |  |
| --- | --- | --- | --- | --- | --- | --- | --- | --- | --- | --- |
|  | No family history of coronary artery disease | | | | |  |  |  |  |  |
| 2001 | He developed hypertension and started taking antihypertensive agents. | | | | | | |  |  |  |
| 2008 | He developed diabetes mellitus and started taking oral hypoglycemic drugs. | | | | | | |  |  |  |
|  |  |  |  |  |  |  |  |  |  |  |
| Dates | Summaries from Initial and Follow-up Visits | | | | | Diagnostic Testing | | Interventions | | |
| 9/4/2018 | The patients developed chest compression at rest. | | | | | Electrocardiography | | Percutaneous Coronary Intervention to the left descending artery | | |
|  |  |  |  |  |  | Blood Examination | |  |  |  |
|  |  |  |  |  |  | Coronary Angiography | | Medical Therapy | | |
| 24/5/2018 | Chest discomfort on effort remained. | | | | | Electrocardiography | | Percutaneous Coronary Intervention to the right coronary artery | | |
|  |  |  |  |  |  |  |  |  |  |  |
|  |  |  |  |  |  |  |  | Medical Therapy | | |
| 26/5/2018 | He did not develop contrast induced acute kidney injury | | | | | Blood Examination | | Medical Therapy | | |
|  |  |  |  |  |  |  |  |  |  |  |
| 11/9/2018 | He had no more chest discomfort and his renal function was preserved. | | | | | Blood Examination | | Medical Therapy | | |
|  |  |  |  |  |  |  |  |  |  |  |
